# Commentary on “Prevalence of Undiagnosed Hypertension Among Adult Displaced Individuals in Baidoa Camps, Somalia (Preprint)”

**DOI:** 10.2196/71041

**Published:** 2025-06-03

**Authors:** Anonymous

**Keywords:** prevalence, undiagnosed, epidemiology, heart, cardiology, cardiovascular, cross-sectional, survey, questionnaires, hypertension, blood pressure, poverty, sedentary, displaced, refugee, Africa

*This is a peer-review report submitted for the preprint “Prevalence of Undiagnosed Hypertension Among Adult Displaced Individuals in Baidoa Camps, Somalia.” The preprint did not proceed to publication in JMIRx Med. In these cases, JMIRx-branded journals, acting as overlay journals for preprints, may publish peer-review reports as commentaries.*

## Round 1 Review

I commend the author for this study [1] on an important topic. However, here are a few comments to help improve the manuscript.

### Title

1. The title needs some slight changes to improve clarity. For instance, what do you mean by “displaced individuals”? Would you rather state it as “internally displaced persons” or just “adults in Baidoa displacement camps”?
2. Use a uniform font for the title.

### Introduction

1. Ensure a consistent referencing style throughout the manuscript.
2. In the sentence “Over the past few decades, the...,” delete the bracket at the end of the statement.
3. Check the overall grammar of the text throughout the manuscript.
4. Regarding the burden of hypertension, provide more updated statistics on hypertension, using both global and regional data. Ensure a clear linkage and transition between the two because, as it stands right now, the statistics are scattered throughout the introduction, rendering it redundant.
5. Provide more context on the displaced populations and their specific vulnerabilities to hypertension to strengthen the rationale of the study. Discuss the factors therein.
6. The section would benefit from a discussion on the effects of hypertension.
7. Cite studies that have investigated hypertension among displaced populations, if any exist, or state the deficit if none.
8. Discuss any interventions and strategies that have been implemented to tackle the problem of hypertension in these communities and state the possible gaps before your objective.

### Methods

1. Formatting issue: provide a heading for your Methods section.
2. As stated above, there is a need to improve the overall grammar.
3. Provide more detail regarding the inclusion criteria. For instance, was there a specific displacement duration that was considered (ie, the minimum amount of time spent in the camp so far)?
4. Provide a justification for the exclusion criteria.
5. Provide the reference for “The sample size for this study was determined....”
6. Add more detail regarding the validation of the questionnaire. Was it adopted from previous studies? Was it pretested?
7. Add detail on the measurement of blood pressure (BP). Who measured the BPs? Were they trained? How did you deal with white-coat hypertension? What was the interval between the different BP readings?

### Results

1. Again, appropriate headings should be provided. Check the grammar.
2. Provide a more simplified and summarized Results section. For instance, “In this study, we enrolled 240 respondents, with a mean age....”
3. Table 1 is very confusing, especially the frequency and percentage columns. Clearly provide both the frequencies and percentages.
4. Add a key for Figure 2 to give better representation or just integrate the data represented into the text.

### Discussion

1. Restate the objective at the start.
2. Provide a concise summary of key findings.
3. Thoroughly discuss the implications of the factors found to be significantly associated with hypertension.

## Abbreviations

BP: blood pressure

## References

[1] Jayte M (2024). Prevalence of undiagnosed hypertension among adult displaced individuals in Baidoa camps, Somalia. medRxiv.

